# UVA Radiation Is Beneficial for Yield and Quality of Indoor Cultivated Lettuce

**DOI:** 10.3389/fpls.2019.01563

**Published:** 2019-12-06

**Authors:** Yongcheng Chen, Tao Li, Qichang Yang, Yating Zhang, Jie Zou, Zhonghua Bian, Xiangzhen Wen

**Affiliations:** ^1^College of Horticulture, Shanxi Agricultural University, Taigu, China; ^2^Institute of Environment and Sustainable Development in Agriculture, Chinese Academy of Agriculture Sciences, Beijing, China; ^3^School of Animal, Rural and Environmental Science, Nottingham Trent University, Nottingham, United Kingdom

**Keywords:** UVA radiation, plant production, secondary metabolites, indoor cultivation, lettuce

## Abstract

Understanding the wavelength dependence of plant responses is essential for optimizing production and quality of indoor plant cultivation. UVA is the main component of solar UV radiation, but its role on plant growth is poorly understood. Here, two experiments were conducted to examine whether UVA supplementation is beneficial for indoor plant cultivation. Lettuce (*Lactuca sativa* L. cv. "Klee") was grown under mixed blue, red, and far-red light with photon flux density of 237 μmol m^−2^ s^−1^ in the growth room; photoperiod was 16 h. In the first experiment, three UVA intensities with peak wavelengths at 365 nm were used: 10 (UVA-10), 20 (UVA-20), and 30 (UVA-30) μmol m^−2^ s^−1^, respectively. In the second experiment, 10 μmol m^−2^ s^−1^ UVA radiation were given for 5 (UVA-5d), 10 (UVA-10d), and 15 (UVA-15d) days before harvest on day 15, respectively. Compared with control (no UVA), shoot dry weight was increased by 27%, 29%, and 15% in the UVA-10, UVA-20, and UVA-30 treatments, respectively, which correlated with 31% (UVA-10), 32% (UVA-20), and 14% (UVA-30) larger leaf area. Shoot dry weight under the treatments of UVA-5d, UVA-10d, and UVA-15d was increased by 18%, 32%, and 30%, respectively, and leaf area was increased by 15%–26%. For both experiments, UVA radiation substantially enhanced secondary metabolites accumulation, e.g. anthocyanin and ascorbic acid contents were increased by 17%–49% and 47%–80%, respectively. Moreover, plants grown under the UVA-30 treatment were stressed, as indicated by lipid peroxidation and lower maximum quantum efficiency of photosystem II photochemistry (F_v_/F_m_). We conclude that UVA supplementation not only stimulates biomass production in controlled environments, but also enhances secondary metabolite accumulation.

## Introduction

Light-emitting diodes (LEDs) are a suitable light source for plant growth (Bantis et al., 2018; Pattison et al., 2018). For a long time, light spectra for plant growth have primarily been selected based on the quantum yield of photosynthesis as measured by McCree (1972). Therefore, red and blue light are considered essential, as they play pivotal roles for leaf photosynthetic functioning (Hogewoning et al., 2012). However, the classic study of McCree (1972) had its limitations, because it was conducted with single leaves and on a short time scale that did not take spectral effects on leaf development into account. Apart from leaf photosynthesis, plants also utilize many photoreceptors that sense specific wavelengths to direct their growth (Heijde and Ulm, 2012; Luo and Shi, 2018). Ultraviolet (UV) radiation is an important component of solar radiation. There are many studies concerning plant responses to UVB (280–315 nm) due to the function of the UVB photoreceptor UVR8, whose function has been clearly elucidated (Wu et al., 2012; Wargent and Jordan, 2013). While UVA (315–400 nm) accounts for ∼95% of solar UV radiation at sea level (Gerhardsson, 2003), there is no conclusive evidence to show that UVA has its own photoreceptor, similar to that of UVB. Instead, UVA can be absorbed by blue light photoreceptors, such as phototropins and cryptochromes (Casal, 2013). However, plant responses to UVA, mediated by these photoreceptors, have so far been poorly investigated (Verdaguer et al., 2017). Thus, the function of UVA in affecting plant growth and development, for example in indoor plant cultivation, is still unclear.

Plant growth is largely determined by morphological and physiological processes that are considerably regulated by the prevailing light conditions (Hernández and Kubota, 2014; Kaiser et al., 2019). UVB consistently leads to a compact phenotype (Ruhland and Day, 2000; Searles et al., 2001; Robson et al., 2015), while a number of studies reported that UVA stimulates leaf size and biomass production (Tezuka et al., 1993; Bernal et al., 2013; Bernal et al., 2015). An earlier study from our lab also reported that UVA supplementation resulted in a larger leaf area and greater stem length in tomato seedlings, thereby facilitating better light interception and accelerating biomass production (Kang et al., 2018). However, other authors showed that UVA can inhibit leaf area expansion and retard biomass accumulation (Newsham et al., 1999; Nigel et al., 2005; Zhang et al., 2014). Moreover, regardless of the direction of plant growth in response to UVA, it is also unclear whether such a response is dose-dependent (Verdaguer et al., 2017).

Improving crop nutritional qualities is an often-pursued endpoint of plant biology. UV radiation is generally considered to be an abiotic stress (Albert et al., 2011; Neugart and Schreiner, 2018), and like some other abiotic stresses promotes the accumulation of secondary metabolites (Ibdah et al., 2002; Neugart and Schreiner, 2018). However, UV radiation may also result in the accumulation of reactive oxygen species (ROS) in plant tissues (Garg and Manchanda, 2009), which may lead to membrane lipid peroxidation and protein degradation. Plants have developed mechanisms to scavenge ROS, such as antioxidative enzymes [e.g. superoxide dismutase (SOD) and catalase (CAT)]. Some antioxidants, such as total phenol and flavonoids, could be rapidly synthesized to prevent cell damage caused by ROS (Garg and Manchanda, 2009). Previous studies have reported that UVA exposure induces flavonoid accumulation, which plays a significant role in UVA screening and as antioxidant (Kotilainen et al., 2008; Kotilainen et al., 2009).

To our knowledge, rigorous investigations using narrow band UVA radiation with different dosage, and its effects on plant growth and secondary compound accumulation in controlled environments are scarce. This study aims at investigating whether UVA supplementation has positive effects on plant growth. Lettuce was used, as it is one of the most popular and suitable species for indoor cultivation. The study was conducted in an environmentally controlled growth room with LED lighting sources. We firstly explored the effects of UVA intensity on lettuce growth and the accumulation of secondary metabolites. Then, we investigated the function of the duration of UVA exposure regulating plant growth and secondary metabolites. From these results, we expect to provide guidance and information for light recipe optimization of indoor farming as well as provide insights into UVA effects on plant growth and development.

## Materials and Methods

### Plant Material and Growth Conditions

Lettuce (*Lactuca sativa* L. cv. "Klee") seeds were sown in plastic germination trays filled with sponge cube blocks (3×3×3 cm) and tap water. One seed was sown per cube. Seeds were germinated in a growth chamber where temperature was 20°C, relative humidity was 65%∼70%. The first three days were fully dark, thereafter, the photosynthetic photon flux density (PPFD) was 100 µmol m^−2^ s^−1^ with photoperiod of 16 h. Upon unfolding of the second leaf (2 weeks after sowing), seedlings were transferred to an environmentally-controlled growth room for treatments. Plants were cultivated in a customized liquid culture hydroponic system with a density of 39 plants per m^2^. In the growth room, CO_2_ partial pressure was close to ambient, day/night temperature was 23/21°C, and relative humidity was 65%∼70%. Photoperiod was 16 h continued from 06:00 to 22:00. Modified Hoagland nutrient solution (pH ≈ 5.8; EC ≈ 1.6 dS m^−1^) was applied for plant cultivation, which was circulated in the system on a daily basis.

**Figure 1:**
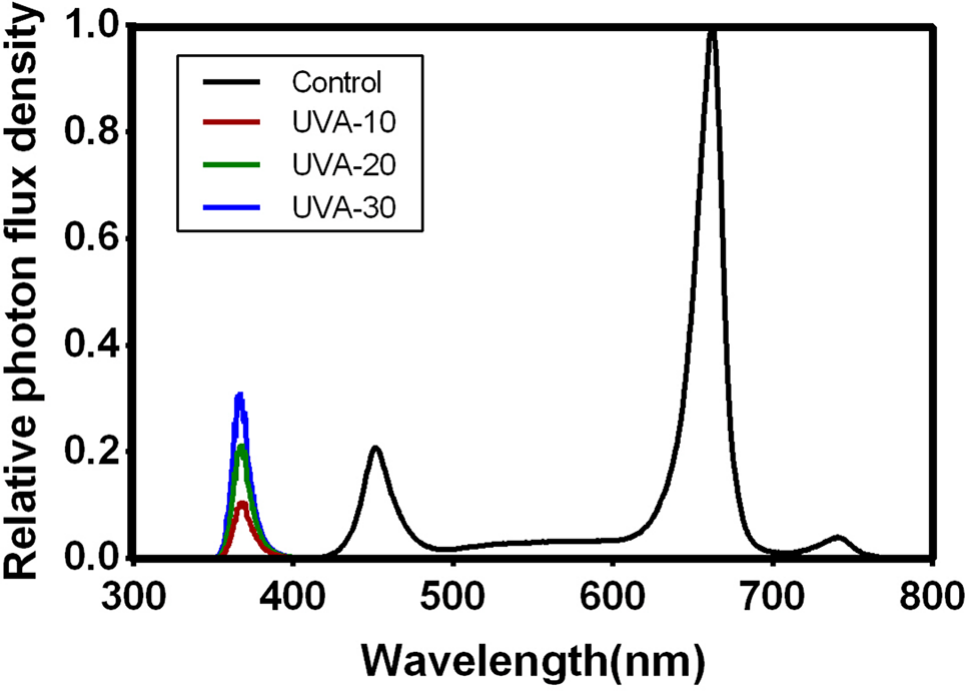
Relative photon flux density in the four treatments. The spectral distribution was measured by a spectrometer (AVANTES 2500, The Netherlands).

In the growth room, two cultivation frames were fixed, and each frame was equally divided into three layers. Dimensions of each frame were: 130 cm length × 70 cm × width 180 cm height. This resulted in six individual cultivation units in the room. The upper four cultivation units were used in this study. To avoid light contamination, opaque black-white plastic films were covered around the cultivation unit. Two ventilation fans (12V, 0.90A) were installed in each units to ensure uniform air circulation. LED light tubes (iGrowLite Co. Ltd, Guangzhou, China) were installed 50 cm above the growth plate. Light intensity and spectra (**Table 1**; **Figure 1**) were monitored using a spectroradiometer (Avaspec-2048CL, Avates, Apeldoorn, The Netherlands), 10 cm above the growth plate. Light intensity was in the range of commercial indoor production facilities (150–250 µmol m^−2^ s^−1^). In this study, we carried out two separate experiments in the same growth room.

**Table 1:** The photon flux density (PFD) at different wavebands of the four treatments.

Treatment	UVA^1^(µmol m^−2^ s^−1^)	PAR^2^(µmol m^−2^ s^−1^)	FR^3^(µmol m^−2^ s^−1^)	Total incident PFD (µmol m^−2^ s^−1^)	DLI^4^(mol m^−2^)	UVA/PAR^5^ (%)
Control	0	230	7	237	13.65	0
UVA-10	10	230	7	247	14.23	4.35
UVA-20	20	230	7	257	14.80	8.70
UVA-30	30	230	7	267	15.38	13.05

^1^UVA radiation, 315–400 nm. ^2^Photosynthetic active radiation, 400–700 nm. ^3^Far-red radiation, 700–750 nm. ^4^Daily light integral, 315–750 nm. ^5^UVA/PAR was 7%–8% in natural sunlight measured in Beijing.

In the first experiment, three UVA intensities of 10, 20, and 30 µmol m^−2^ s^−1^ with peak wavelength at 365 nm were used in three of the cultivation units, respectively. These are referred to as UVA-10, UVA-20, and UVA-30 (**Table 1**). A fourth treatment, without supplemental UVA, is considered to be the control. UVA radiation was emitted by UVA LED light tubes that connected with a light modulator (iGrowLite Co. Ltd). During the experiment, four batches of lettuce were grown in succession, each batch for 13 days. The treatment position was randomly switched whenever new cultivation started.

In the second experiment, two batches of lettuce were grown in succession, and plants were grown for 15 days. UVA radiation (10 µmol m^−2^ s^−1^) was supplemented at day 0, 5, and 10, respectively, in three of the cultivation units, meaning that UVA radiation was supplemented for 15 (UVA-15), 10 (UVA-10), and 5 (UVA-5) days before harvest on day 15. Again, a fourth treatment without UVA was added as control.

### Destructive Measurements

Destructive measurements were conducted 13 days after start of treatments in the first experiment, and 15 days after start of treatment in the second experiment. Plants were harvested at a younger stage than those in commercial cultivation, in order to reduce effects of shading between larger plants. Four plants from each cultivation unit were randomly selected at each batch of plant cultivation, i.e. 16 plants were harvested per treatment for the first experiment, and 8 plants were harvested per treatment for the second experiment. Leaves, stems, and roots were separated. Leaf area was measured with a leaf area meter (LI-3100C, Li-Cor Biosciences, Lincoln, Nebraska, USA). After fresh weight determination, leaves, stems, and roots were dried in an oven at 80°C for 48 h. Specific leaf area was calculated by dividing the leaf area by leaf dry weight.

### Gas Exchange and Chlorophyll Fluorescence Measurements

Gas exchange and chlorophyll fluorescence were measured in the second batch of plants in the first experiment, on day 11 after start of treatments, between 8:30 and 16:30. Five plants from each cultivation unit were randomly selected, and measurements were performed on the uppermost fully expanded leaves. Measurements were performed with the LI-6400XT photosynthesis system (Li-Cor Biosciences) with the leaf chamber fluorometer (Li-Cor Part No. 6400-40, area 2 cm^2^), in which a mixture of red (90%) and blue (10%) LEDs with peak intensities of 635 and 465 nm, respectively, was provided. The starting PPFD was 250 µmol m^−2^ s^−1^, followed by 1500 µmol m^−2^ s^−1^. At each PPFD, measurements were taken when the photosynthetic rate reached steady state (∼10 min). During measurements, CO_2_ concentration was 450 µmol mol^−1^, leaf temperature was 22°C, leaf-to-air vapor pressure deficit (VPD_leaf-air_) was maintained between 0.7 and 1.0 kPa, and the flow rate of air through the system was 500 µmol s^−1^.

To assess treatment effects on chlorophyll fluorescence, leaves were dark-adapted with a leaf clip holder (DLC-8) for 1 h prior to measurements. Thereafter, the selected leaf was measured with a portable chlorophyll fluorometer (PAM-2100, Walz, Effeltrich, Germany). Red light was used for measuring light and for saturating flashes. Minimal (F_0_) and maximal (F_m_) fluorescence were recorded to determine the maximum quantum efficiency of photosystem II photochemistry (F_v_/F_m_).

### Leaf Biochemical Components and Enzyme Activity Determination

One day before destructive measurements, uppermost fully expanded leaves were collected in vials, flash-frozen in liquid nitrogen, and transferred to a freezer (−80°C) for storage. These samples were later used to determine leaf biochemical components and enzyme activity.

#### Pigment Concentrations

Fresh leaf samples (0.1 g) were submerged in 10 mL 95% ethanol at room temperature for 24 h in darkness. The absorbance of the extract was measured at 665, 649, and 470 nm using a UV-Vis spectrophotometer (UV-1800, Shimadzu, Japan). Chlorophyll and carotenoid concentrations were calculated using equations derived by Wellburn (1994).

#### Soluble Sugar and Protein Content 

Leaf soluble sugar content was determined with the phenol–sulfuric acid method from DuBois et al. (1956). Fresh leaf samples (0.1 g) were ground and extracted with boiling water and subsequently filtered. Filtrate (0.125 mL ) was collected and mixed with 0.375 mL distilled water, 0.25 mL phenol (1 M), and 1 mL concentrated sulfuric acid (98%) for color reaction (30 min). Thereafter, the absorbance of the extraction at 485 nm was measured with a UV-Vis spectrophotometer (UV-1800). Leaf soluble protein content was determined following Snyder and Desborough, (1978). Fresh samples (0.1 g) were ground and extracted with distilled water and subsequently filtered. Filtrate (0.1 mL) was collected and mixed with 1 mL Coomassie Brilliant Blue G-250 solution for 2 min. The absorbance of the extract at 595 nm was measured with a UV-Vis spectrophotometer (UV-1800).

#### Phenolic and Flavonoid Contents 

Fresh leaf samples (0.1g) were ground in a mortar and pestle with liquid nitrogen, and were then extracted with 1 mL 80% aqueous methanol in an ultrasonic bath for 10 min, and were then centrifuged for 10 min at 15,000 rpm. The contents of total phenolic and flavonoid were determined by using the Folin-Ciocalteu assay and aluminum chloride colorimetric assay, respectively, following Khanam et al. (2012). The absorbance against prepared reagent blank was determined using a microplate reader (Infinite 200 PRO, TECAN, Switzerland). For total phenolic content, gallic acid was used as the standard reference and gallic acid equivalent (GAE) was expressed as µg GAE/100 g fresh mass. For total flavonoid content, Rutin was used as the standard reference and RUE was expressed as µg rutinum equivalent (RUE)/100 g fresh mass.

#### Anthocyanin Content

Anthocyanin content was determined by the pH differential method following Giusti and Wrolstad (2001). Fresh leaf samples (0.1 g) were extracted with 1% (v/v) HCl-methanol, and subsequently incubated for 24 h at 4°C in darkness. The mixture was centrifuged (13,000×g, 15 min, 4°C), and 0.4 mL supernatant was collected and divided equally into two copies, one with potassium chloride buffer (0.025 M, pH 1.0), the other with sodium acetate buffer (0.4 M, pH 4.5); the dilution factor for the sample was 15. Samples were equilibrated for 15 min, after which absorbance of each dilution was measured at 530 and 700 nm against a blank filled with distilled water using a UV-Vis spectrophotometer (UV-1800).

#### Malondialdehyde (MDA) Content

MDA content was determined using the method of Hodges et al. (1999). Fresh leaf samples (0.1 g) were homogenized in 1 mL cold (4°C) 10% (w/v) trichloroacetic acid. The homogenate was centrifuged (13,000×g, 15 min, 4°C), 0.5 mL supernatant was collected for adding 1 mL 6% (w/v) thiobarbituric acid solution, which was boiled at 100°C for 20 min. The supernatant was cooled to room temperature and then centrifuged (15,000×g) for 10 min. The absorbance of the extract at 450, 532, and 600 nm was measured using a UV-Vis spectrophotometer (UV-1800).

#### SOD and CAT Activities

Fresh leaf samples (0.1 g) were homogenized with 1 mL ice-cold extraction buffer (25 mM phosphate buffer, pH 7.8) using a commercial assay kit (Comin biotechnology Co. Ltd. Suzhou, China) containing 1% polyvinylpyrrolidone. The homogenized material was centrifuged (18,000 ×g 4°C) for 30 min. Supernatant (1 mL) was collected to assay the activities of SOD and CAT. SOD activity was determined by the method of Wu et al. (2007) with adaptations, and was assayed by monitoring the inhibition of photochemical reduction of nitro-blue tetrazolium chloride (NBT). A reaction mixture consisting of 0.1 mL methionine (200 mM), 0.1 mL NBT (2.25 mM), 0.1 mL EDTA (3 mM), 0.1 mL riboflavin (60 mM), 0.5 mL phosphate buffer (25 mM), and 0.1 mL enzyme preparation in a total volume of 1.1 mL was used. The mixture was placed under a fluorescent light (4000 lux, 25°C) for 20 min. Absorbance at 560 nm was monitored using a microplate reader (Infinite 200 PRO). CAT activity was determined using the method of hydrogen peroxide (H_2_O_2_) UV absorption adapted from Beers and Sizer (1952), using a commercial assay kit (Comin biotechnology). Enzyme preparation (35 µL) was mixed with 1 mL H_2_O_2_ (0.1 M). The initial absorbance at 240 nm and the absorbance after 1 min at 240 nm was determined using the UV-Vis spectrophotometer (UV-1800).

#### Determination of Superoxide Anion Radical (O_2_−) Generation

O_2_^− ^generation rate was determined with the sulfamate colorimetric method of Elstner and Heupel (1976), using a commercial superoxide anion radical assay kit (Leagene Biotechnology Co. Ltd. Beijing, China). Fresh leaf samples (0.1 g) were homogenized by 1 mL cold O_2_^−^ lysis buffer. The homogenized material was centrifuged (10,000 × g 4°C) for 10 min. 0.5 mL supernatant was collected and mixed with 0.5 mL hydroxylamine hydrochloride (1 mM) and incubated for 1 h at 25°C. Then, 20 mg of activated carbon was added to the mixture and immediately centrifuged (10,000×g, 25°C) for 5 min. The activated carbon was added to adsorb macromolecular complexes (e.g. anthocyanin) to avoid pigment interference. Supernatant (0.5 mL) was mixed with p-aminophenylsulfonic acid (17 mM, 0.5 mL) and α-naphthylamine (7 mM, 0.5 mL), and incubated for 20 min at 30°C. Thereafter, the absorbance at 530 nm was measured with a microplate reader (Infinite 200 PRO).

#### Ascorbic Acid Content 

Ascorbic acid content was determined as in Spínola (2012) and Campos (2009) with adaptations. Fresh leaf samples (0.1g) were homogenized by 1 mL cold extraction buffer (3% MPA + 8% acetic acid + 1 mM EDTA solution). The homogenate was centrifuged (15,000×g, 4°C) for 10 min, 50 µL supernatant was collected and mixed with 10 µL dithiothreitol (750 mM), 190 µL Tris buffer (200 mM), and 50 µL sulfuric acid (0.4 M), which were incubated at 28°C for 30 min. The mixture was filtered through 0.22 µm PTFE filters (Jinteng Co. Ltd., Tianjin, China). The analysis was performed using the Acquity UPLC system (Waters Corp, USA) with an Acquity UPLC^®^ HSS T3 (2.1 × 100 mm, 1.8 µm, Waters) column, equipped with a Waters Acquity UPLC photodiode array (Waters Corp, USA) detection system. A standard solution was formulated using L-ascorbic acid, the mobile phase was 0.1% (v/v) formic acid and the velocity of flow was 0.25 mL/min.

### Statistics

Data were analyzed by one-way ANOVA in randomized blocks, using SPSS 23 (SPSS Inc., Chicago, IL). The experimental replicate was regarded as a blocking factor. Data were first tested for normality (Shapiro–Wilk test) and homogeneity of variances (Levene’s test). Subsequently, least significant differences of treatment effects were determined (*P* = 0.05).

## Results

### Biomass and Morphology in Response to UVA Intensities

**Figure 2:**
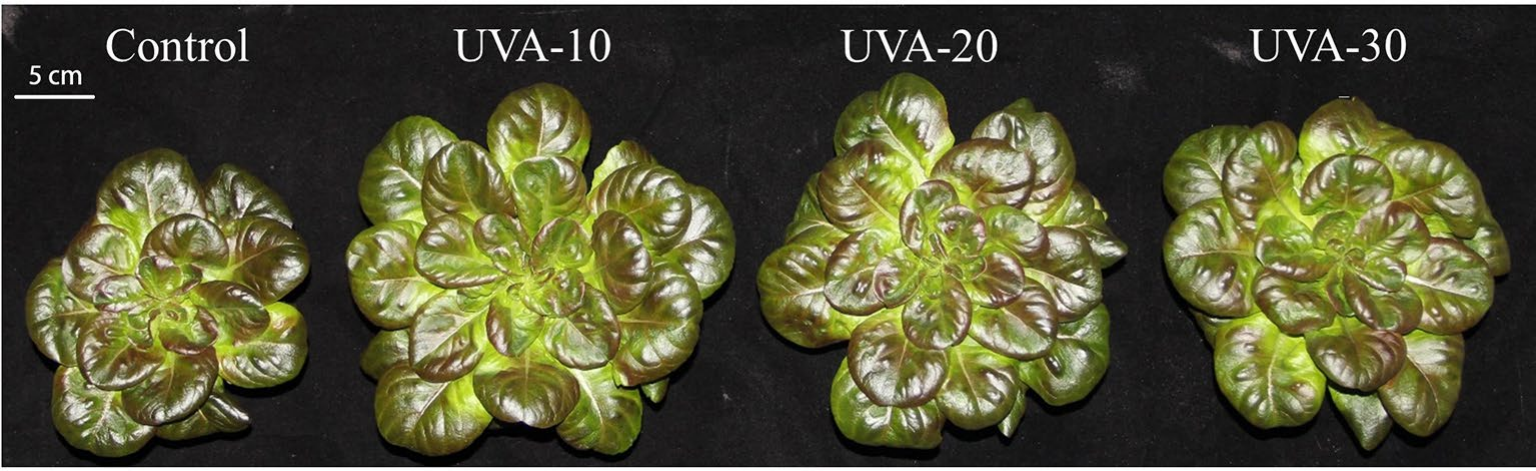
Lettuce plants grown under four different light conditions 13 days after treatment. UVA-10, UVA-20, and UVA-30 represent treatments with 10, 20, and 30 µmol m^−2^ s^−1^ UVA radiation, respectively.

UVA supplementation significantly stimulated biomass production of indoor cultivated lettuce (**Table 2**). Specifically, adding 10, 20, and 30 µmol m^−2^ s^−1^ UVA radiation resulted in 27% (UVA-10), 29% (UVA-20), and 15% (UVA-30) higher shoot dry weight, respectively, compared with that of control. Leaf area was increased by 31%, 32%, and 14% in the UVA-10, UVA-20, and UVA-30 treatments, respectively (**Figure 2**; **Table 2**). Moreover, leaf number was also stimulated by UVA radiation (11%–18%). Specific leaf area, shoot/root ratio, and shoot dry mass content were not affected by UVA (**Table 2**).

**Table 2:** Plant growth and morphology in response to different UVA intensities.

Treatment	Control	UVA-10	UVA-20	UVA-30
Shoot fresh weight (g plant^−1^)	31.8 ± 1.8c	41.6 ± 2.4a	41.7 ± 1.5a	36.5 ± 2.4b
Shoot dry weight (g plant^−1^)	1.77 ± 0.10c	2.24 ± 0.11a	2.29 ± 0.09a	2.03 ± 0.12b
Leaf area (cm^2^ plant^−1^)	752.1 ± 51.7c	981.6 ± 58.3a	989.8 ± 40.7a	855.9 ± 57.6b
Leaf number	30.2 ± 1.4c	34.4 ± 1.3ab	35.5 ± 1.1a	33.5 ± 1.5b
Specific leaf area (cm^2^ g^−1^)	402.7 ± 15.9	435.1 ± 18.6	428.9 ± 15.1	404.4 ± 15.0
Shoot/root ratio	6.4 ± 0.3	6.4 ± 0.4	6.4 ± 0.3	6.6 ± 0.9
Shoot dry mass content (%)	5.6 ± 0.2	5.4 ± 0.2	5.5 ± 0.1	5.6 ± 0.2
Leaf light absorption (%) (400–700 nm)	93.2 ± 0.3	92.6 ± 0.8	92.1 ± 0.8	91.4 ± 1.1

Data represent mean ± SE (n = 16). Means followed by different letters within one row differ significantly (P < 0.05) as established by the least significant difference test.

### Leaf Photosynthetic Properties in Response to UVA Intensities

**Figure 3:**
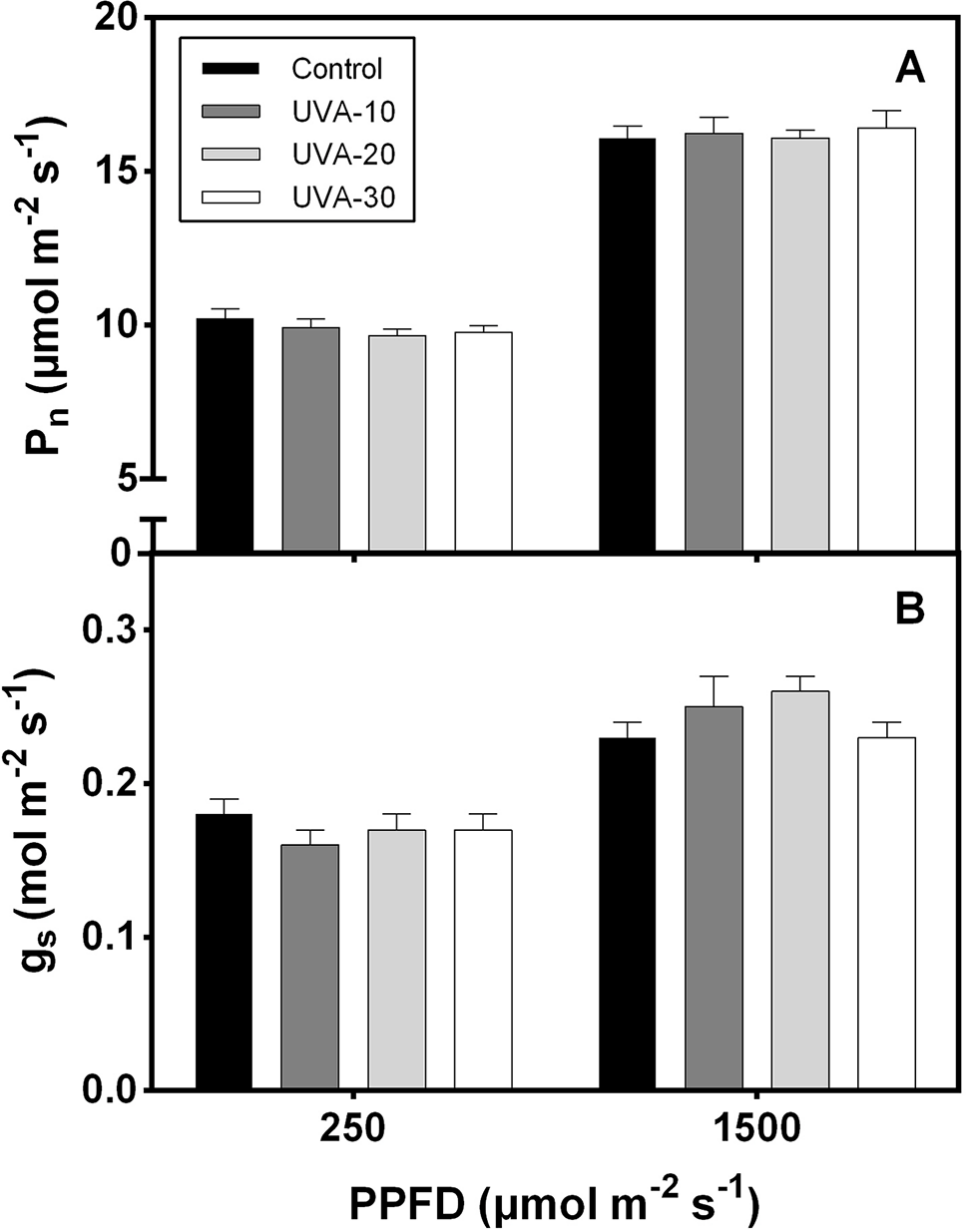
**(A) **Net leaf photosynthetic rate (P_n_) and **(B)** stomatal conductance (g_s_) of lettuce leaves in response to low (250 µmol m^−2^ s^−1^) and high (1500 μmol m^−2^ s^−1^) photosynthetic photon flux density (PPFD). Error bars show ± SE (n = 5).

**Figure 4:**
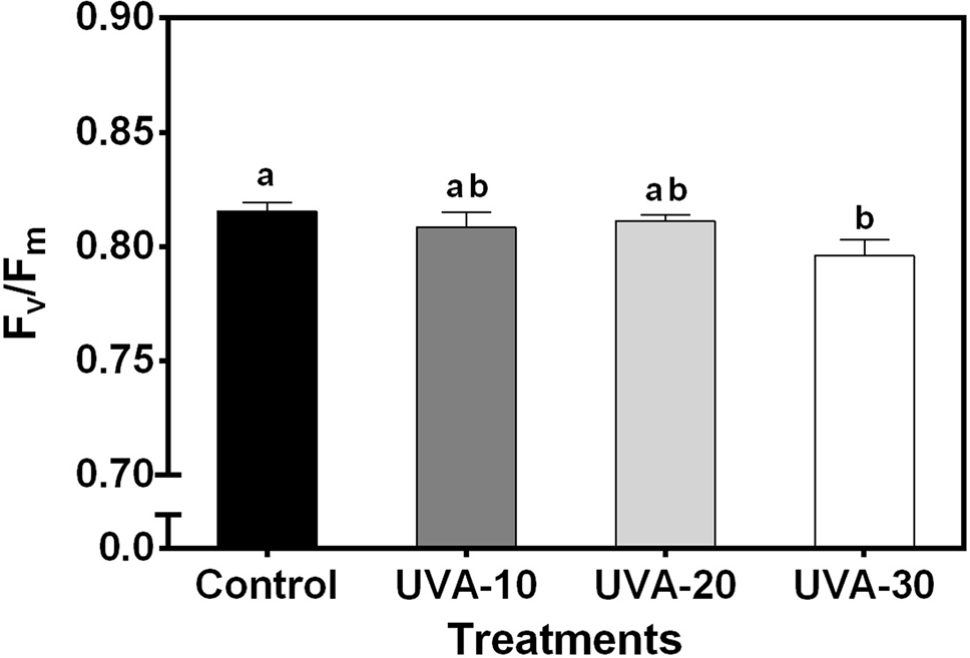
Effects of UVA on maximum quantum efficiency of photosystem II photochemistry (F_v_/F_m_) of lettuce leaves. Error bars show ± SE (n = 5). Letters show statistically significant differences (*P < 0.05*).

When measured under an identical spectrum (90% red +10% blue, no UVA), net leaf photosynthetic rate and stomatal conductance were unaffected by UVA at low (250 µmol m^−2^ s^−1^, close to the growth irradiance) and high (i.e. 1500 µmol m^−2^ s^−1^) PPFD (**Figure 3**). Growth under UVA-30 led to a slight decrease in F_v_/F_m_ compared with control, while under UVA-10 and UVA-20 F_v_/F_m_ was unchanged (**Figure 4**).

### Leaf Biochemical Components, Pigmentation, and Antioxidants in Response to UVA Intensities

Total phenolic and flavonoid contents were increased under UVA-20 and UVA-30 treatments (**Table 3**). Leaves grown under UVA showed higher anthocyanin content, in particular under UVA-30 (increased by∼50%), compared to the control. Ascorbic acid content was 60%–80% higher than that of control in the three UVA intensity treatments (**Table 3**). O_2_^−^ generation rate was increased under UVA-20 and UVA-30, while there was no difference between UVA-10 and control. Membrane lipid peroxide, measured as MDA content, was increased under UVA-30 compared with all other treatments. Furthermore, UVA increased the activities of SOD and CAT (**Table 3**). Also, plants grown under UVA had increased soluble sugar and protein contents. No significant differences were detected for chlorophyll and carotenoid contents (**Table 3**).

**Table 3:** Leaf biochemical components and antioxidant enzyme activities in response to different UVA intensities.

Treatment	Control	UVA-10	UVA-20	UVA-30
Total phenolic (mg GAE g^−1^·FW)	1.80 ± 0.06b	1.84 ± 0.07b	1.99 ± 0.06ab	2.12 ± 0.08a
Total flavonoids (mg RUE g^−1^·FW)	6.0 ± 0.3b	7.3 ± 0.4ab	8.4 ± 0.6a	8.9 ± 0.6a
Anthocyanin (mg 100 g^−1^·FW)	16.0 ± 0.8c	22.1 ± 0.8ab	20.5 ± 0.6b	23.9 ± 1.1a
Ascorbic acid (μg g^−1^·FW)	204.8 ± 9.5c	340.2 ± 9.5b	368.9 ± 4.9a	329.8 ± 11.5b
Total MDA (nmol g^−1^·FW)	3.14 ± 0.14b	3.69 ± 0.17b	3.53 ± 0.25b	4.40 ± 0.19a
O_2_^−^ generation rate (nmol min^−1^ g^−1^·FW)	19.5 ± 0.5c	20.5 ± 0.6c	22.8 ± 0.7b	24.6 ± 0.6a
SOD activity^1^ (U min^−1^ g^−1^·FW)	169.0 ± 2.3b	181.7 ± 2.5a	180.5 ± 2.3a	186.9 ± 2.3a
CAT activity^2^ (U min^−1^ g^−1^·FW)	328.3b ± 3.4	366.9 ± 4.1a	374.8 ± 3.9a	362.8 ± 2.6a
Chl (a + b) (mg m^−2^)	612.7 ± 34.0	662.5 ± 34.5	662.2 ± 29.5	682.4 ± 39.2
Carotenoid (mg m^−2^)	155.6 ± 9.0	166.6 ± 8.9	167.3 ± 7.4	172.4 ± 9.7
Soluble sugar content (mg g^−1^·FW)	15.7 ± 0.5c	19.8 ± 0.6a	17.7 ± 0.6b	17.7 ± 0.8b
Total soluble protein (mg g^−1^·FW)	5.1 ± 0.3c	6.3 ± 0.3a	6.1 ± 0.3ab	5.8 ± 0.3b

Data represent mean ± SE (n = 16). Means followed by different letters within one row differ significantly (P < 0.05) as established by the least significant difference test.

^1^50% of the photoreduction of nitroblue tetrazolium (NBT) is used as an enzyme activity unit.

^2^1 nmol of H_2_O_2_ degradation per minute is defined as an enzyme activity unit.

### Biomass and Morphology in Response to Different Durations of UVA Exposure

Compared with the control, UVA exposure increased biomass production (**Table 4**). Specifically, shoot dry weight was increased by 18%, 32%, and 30% under UVA-5d, UVA-10d, and UVA-15d, respectively. Leaf area was increased by 15%–26% in the UVA duration treatments, compared to control (**Table 4**).

**Table 4:** Plant production and morphological properties in response to different durations of UVA exposure.

Treatment	Control	UVA-5d	UVA-10d	UVA-15d
Shoot fresh weight (g plant^−1^)	39.1 ± 1.5c	46.1 ± 1.2b	50.8 ± 1.0a	49.9 ± 1.4a
Shoot dry weight (g plant^−1^)	2.11 ± 0.08c	2.48 ± 0.10b	2.79 ± 0.09a	2.75 ± 0.12a
Leaf area (cm^2^ plant^−1^)	908.4 ± 34.8c	1047.1 ± 17.4b	1146.6 ± 16.4a	1105.1 ± 26.5ab
Leaf number	36.4 ± 0.9b	40.1 ± 0.7a	40.6 ± 1.1a	40.8 ± 0.9a

Data represent mean ± SE (n = 8). Means followed by different letters within one row differ significantly (P < 0.05) as established by the least significant difference test.

### Leaf Biochemical Components and Antioxidants in Response to Different Durations of UVA Exposure

With 10 µmol m^−2^ s^−1^ supplemental UVA, anthocyanin content significantly increased under different durations of UVA exposure. Five days of UVA exposure (UVA-5d) resulted in ∼17% higher anthocyanin content compared to the control (**Table 5**). The ascorbic acid content was increased by 47%–63% under the three UVA durations, without a difference between these treatments. For total phenolic, a significant difference was detected only in the UVA-10d treatment (**Table 5**), although there was a trend for an increase under the other UVA treatments as well, compared with control. Total soluble sugar content was increased by 22%, 42%, and 37% under UVA-5d, UVA-10d, and UVA-15d, respectively, compared with control (**Table 5**).

**Table 5:** Leaf biochemical components and antioxidant enzyme activities in response to different durations of UVA exposure.

Treatment	Control	UVA-5d	UVA-10d	UVA-15d
Total phenolic (mg GAE g^−1^·FW)	1.62 ± 0.05b	1.97 ± 0.11ab	2.05 ± 0.13a	1.97 ± 0.14ab
Total flavonoids (mg RUE g^−1^·FW)	7.9 ± 0.5	8.7 ± 1.0	8.5 ± 0.8	8.8 ± 0.8
Anthocyanin (mg 100 g^−1^·FW)	20.3 ± 0.4c	23.8 ± 0.2b	23.9 ± 0.3b	25.2 ± 0.6a
Ascorbic acid (μg g^−1^·FW)	208.4 ± 16.2b	307.3 ± 15.4a	340.1 ± 7.7a	322.4 ± 14.8a
Soluble sugar content (mg g^−1^·FW)	16.6 ± 0.6c	20.2 ± 0.8b	23.6 ± 0.6a	22.7 ± 0.4a

Data represent mean ± SE (n = 8). Means followed by different letters within one row differ significantly (P < 0.05) as established by the least significant difference test.

## Discussion

Plant responses to UVA radiation are so far poorly understood and the available literature often contains contradictory information (Verdaguer et al., 2017; Neugart and Schreiner 2018). This may partly be due to limitations of past experimental approaches that suffered from a paucity of available LEDs. Consequently, many of these experiments were performed outdoors, where natural UV-exposure was modulated using cut-off filters (Martz et al., 2007; Morales et al., 2009; Kataria et al., 2013). These past approaches make it difficult to fully take into account all the possible side effects such as interaction with UVB and PAR. Due to rapid advances in LEDs that allow for much finer control of spectrum and intensity of UVA, we now are able to expose plants to well-defined UVA intensities and durations under fully controlled conditions. Here, we show that adding UVA in a controlled environment not only stimulates biomass production (**Tables 2** and **4**), but also improves the nutritional quality of lettuce (**Tables 3** and **5**). In natural sunlight, the ratio of UVA/PAR is 7%–8% (measured in Beijing). While a UVA/PAR ratio of 4.35% (i.e. UVA-10) substantially improved lettuce production and quality, a further increase to 8.7% (i.e. UVA-20) and 13.05% (i.e. UVA-30) did not have additional effects (**Tables 2** and **3**). This indicates that in commercial indoor lettuce cultivation, the ratio of UVA/PAR higher than the natural sunlight is not necessary.

### UVA Increases Biomass Production, But Only to an Optimum

Plant biomass production is highly correlated with growth conditions. We showed that UVA supplementation resulted in 15%–29% higher shoot dry weight (**Table 2**). This is in agreement with several previous studies that have reported that UVA has a stimulatory effect on biomass accumulation (Tezuka et al., 1993; Lee et al., 2014; Bernal et al., 2015). However, others reported an inhibitory effect of UVA on plant growth (Krizek et al., 1997; Krizek et al., 1998; Kataria et al., 2013). Verdaguer et al. (2017) proposed that such variable responses might be caused by interaction with other environmental factors (Bernal et al., 2013), as well as dependent on the species or even on the genotype (Kataria and Guruprasad, 2012). Moreover, differences in UVA peak wavelength applied in different studies may also play a role for this controversy.

Plant biomass production is to a large extent mediated by the plant light interception and daily light integral (Li et al., 2014). In this study, the daily light integral in the three UVA treatments was 4.2%–12.7% higher than in the control (**Table 1**). Although in horticulture, it is often assumed that roughly, 1% additional light results in 1% additional growth and production (Marcelis et al., 2006), this apparently does not agree with the current study, because the extra light here is UVA, which has extremely low photosynthetic quantum yields in comparison with visible radiation (McCree, 1972). Thus, the increase in leaf area and plant biomass seen under UVA is unlikely to have resulted from a direct increase of photosynthesis due to a hypothetical larger availability of radiation that could be used for photosynthesis.

Light interception is dependent on plant architecture (Sarlikioti et al., 2011). Therefore, the increase in leaf area due to UVA exposure (14%–32%) might play a pivotal role for contributing to increased biomass, because the larger leaf area mean higher light interception, which is one of the driving forces behind plant photosynthesis (Sarlikioti et al., 2011). On the other hand, light is a major determinant of plant architecture, and this process is mediated by a range of photoreceptors (Casal, 2013). For instance, UVB consistently inhibits stem elongation and leaf expansion through UVR8 (Heijde and Ulm, 2012). Consistent with our observation, previous studies reported that supplemental UVA substantially increased rosette diameter of *Arabidopsis thaliana* (Jansen and Biswas, 2012) and flag leaf area in some *Sorghum bicolar* varieties (Kataria and Guruprasad, 2012). In this context, the underlying mechanisms of UVA affecting plant morphology are distinct from the function of UVB on plant morphology.

Although data from previous studies are insufficient for generating a dose response of UVA effects on plant growth (Verdaguer et al., 2017), we showed that biomass production and leaf area of indoor cultivated lettuce were not linearly increased with the intensity of UVA. On the contrary, they were inhibited at 30 µmol m^−2^ s^−1^ in comparison with 10 µmol m^−2^ s^−1^. This indicates that growth of indoor cultivated lettuce may have a saturating response to UVA. For further validation, we carried out another experiment in which treatments had the same UVA intensity but different durations of UVA exposure, which showed that UVA exposure for 10–15 days resulted in similar biomass production and leaf area, again indicating a saturating response. More treatments with different UVA intensities or exposure durations would be useful for figuring out the specific saturating UVA dose.

### UVA Does Not Downregulate Leaf Photosynthetic Capacity, But Photoinhibits Leaves At High Intensity

UVA radiation has been considered as a damaging factor for photosynthesis (Vass et al., 2002; Tyystjarvi 2008). However, our data show that plants grown under the three UVA intensity treatments had similar leaf photosynthetic rates as the control (**Figure 3**). These data do not reflect short-term effects of UVA treatments on photosynthesis under their respective growth condition as they were measured without UVA. Rather, these data reflect possible long-term (acclimation) effects of these treatments on the photosynthetic apparatus; the data indicate that leaf photosynthetic activity and stomatal conductance (measured under 250 and 1500 µmol m^−2^ s^−1^) were not downregulated by UVA exposure.

Leaves grown under UVA-30 exhibited signs of photoinhibition (lower F_v_/F_m_, **Figure 4**) that did not affect leaf photosynthetic rates (**Figure 3A**). This indicates that plants grown under this treatment were experiencing harmful effects of UVA on photosystem II protein D1 turnover (Vass et al., 2002). On the other hand, a previous study reported that UVA can enhance photosynthetic rates when supplemented at non-saturating levels of visible light, and this effect was mainly caused by UV-induced violet-blue-green fluorescence that is harvested by photosynthetic pigments to drive electron transport (Mantha et al., 2001). This stimulating effect was observed under a short-term exposure to UVA (Mantha et al., 2001). It is unclear whether such a mechanism also occurred in the current study. Therefore, further work is needed to assess whether the above-mentioned enhancement effect occurs in the plants that are developed under UVA radiation.

### UVA Promotes Secondary Metabolite Production

UV radiation has traditionally been considered as environmental stressor that can lead to the generation of ROS (Wargent and Jordan, 2013). We also observed that plants grown under UVA treatments had higher superoxide anion radical (O_2_^−^) generation rate, in particular under the UVA-30 treatment (**Table 3**). ROS can cause considerable cellular damage through oxidation of lipids, proteins, and DNA (Garg and Manchanda, 2009). Therefore, the UVA induced increase in antioxidant contents act as an effective ROS scavenger (Treutter, 2006). Here we showed that increasing UVA intensity or extending the duration of UVA exposure did not remarkably increase the concentration of total phenolics in lettuce (**Table 3**). This is in line with Verdaguer et al. (2017), who suggested that for understanding the effects of UVA on plant metabolites, attention should be given to the alterations in individual phenolic compounds, rather than the total phenolic content.

Flavonoids are a major group of phenolics, which is often associated with plant responses to UV radiation (Treutter, 2006). Therefore, it is not surprising that lettuce grown under higher intensities of UVA (i.e. UVA-20 and UVA-30) had higher total flavonoid contents than that of control (**Table 3**). Such UVA induced flavonoids could play a significant role both in UVA screening and as antioxidants in order to prevent cell damage caused by UVA (Treutter, 2006). Anthocyanins are important components of flavonoids, and their contents were significantly increased by UVA (**Table 3**), and these results are similar to Lee et al. (2014). Anthocyanins plays a photoprotective role in enhancing plant tolerance to abiotic stresses: for example, they absorb a fraction of yellow/green and ultraviolet wavelengths, and consequently may reduce the damage to photosystem II (Landi et al., 2015). Ascorbic acid also acts primarily as antioxidant (Garg and Manchanda, 2009), and was substantially increased by UVA in our study (**Tables 3** and **5**), consistent with Jeong et al. (2009). Secondary metabolite synthesis requires substrate availability, i.e. photosynthates (Coley et al., 1985). In this context, increased secondary metabolite contents in the UVA treatments might be correlated with higher soluble sugar content (**Tables 3** and **5**).

Plant oxidative protection also depends on antioxidant enzymes (Gill and Tuteja, 2010; Vighi et al., 2017), such as SOD and CAT, which were significantly upregulated by UVA radiation (**Table 3**). These enzymes are indispensable for ROS detoxification (Willekens et al., 1997). To mitigate possible detrimental effects caused by UVA, therefore, plants could maintain a balance between antioxidative protection and ROS production. However, such a balance was likely broken under the UVA-30 treatment, indicated by a significantly higher MDA content (**Table 3**), as MDA is a typical end product of lipid peroxidation and is considered as the first type of oxidative damage (Taylor et al., 2002).

## Conclusion

Supplementing UVA radiation to LED light in a controlled environment induced larger leaf area, thereby facilitating better light interception and substantially increased biomass production. Moreover, UVA radiation also enhanced secondary metabolite accumulation in lettuce. Under high UVA intensity, plants were stressed, as indicated by lipid peroxidation (i.e. higher MDA content) and lower maximum quantum efficiency of photosystem II photochemistry (F_v_/F_m_). Our results suggest that the stimulating effect of UVA on lettuce growth exhibits a saturation response to the UVA dose.

## Data Availability Statement

The datasets generated for this study are available on request to the corresponding author.

## Author Contributions

YC and TL conceived and designed the experiments. YC, YZ and JZ performed the experiments and statistical analysis. YC and TL wrote the manuscript. QY, XW and ZB contributed to manuscript revision, read and approved the submitted version.

## Funding

This work was financially supported by the National Key Research and Development Program of China (2017YFB0403902), the National Natural Science Foundation of China (No. 31501808 and 31872955), and the Central Public-interest Scientific Institution Basal Research Fund (No. BSRF201911).

## Conflict of Interest

The authors declare that the research was conducted in the absence of any commercial or financial relationships that could be construed as a potential conflict of interest.
